# Virtual Reality Hypnoanxiolysis and Analgesia for Emergency Department Needle-Related Procedures: A Prospective Interventional Cohort Pilot Study Implementation

**DOI:** 10.1016/j.mcpdig.2023.05.001

**Published:** 2023-06-30

**Authors:** Donnchadh O’Sullivan, Jason O’Callaghan, Michael Barrett

**Affiliations:** a Department of Emergency Medicine, Children’s Health Ireland, Crumlin, Dublin, Ireland; b The D4 Wellness Clinic, Blackrock, Dublin, Ireland; c Women’s and Children’s Health, School of Medicine, University College, Dublin, Ireland; d Pediatric and Adolescent Medicine, Mayo Clinic, Rochester, MN

## Abstract

We hypothesized that virtual reality–delivered hypnotherapy reduces anxiety (hypnoanxiolysis) and pain (hypnoanalgesia) related to needle procedures. Our primary objective was to assess whether this would result in lower procedural anxiety and/or pain scores from baseline for pediatric patients undergoing nonemergent needle-related procedures. A prospective interventional pilot study of a cohort of 22 patients (aged 8-15 years) who required a needle procedure in the emergency department was conducted, and parents (dyad) were consented. Anxiety and pain measurements were taken before and after the intervention for each dyad. The worst anxiety reflected the maximal anxiety intensity experienced by the participant during the procedure using the Visual Analog Anxiety Scale and, similarly for pain, using the validated pediatric Color Analog Pain Scale. Patients, parents, and physicians completed satisfaction scores. Most of the participants reported a reduction in anxiety during the procedure. The participants’ median maximum procedural pain scores were slightly increased from baseline. Eleven (50%) children and 19 physician and parent reports (85%) stated that the needle intervention was easy or very easy. We described novel implementation of virtual reality hypnotherapy in an emergency setting and achieved anxiety reduction for needle-related procedures in a pediatric cohort.

Children rate needle procedures as their most painful health care experience, with their primary pathology and caregivers rating needles as one of the most distressing aspects of patient care.^1^^,^^2^ The connectivity of pediatric frontal lobe structures with the rest of the brain is not fully mature and, thus, makes it difficult for children to control their fear. This connectivity is important for the control of intense affective states.^3^

Recent reviews and meta-analyses have strongly suggested that virtual reality (VR) is useful for anxiety/distress and pain reduction in a wide variety of medical procedures.^4^, ^5^, ^6^, ^7^ Virtual reality is a relatively new technology that allows an individual to experience and be present in a virtual world. “Presence” has been proposed as a key factor for superior anxiety and pain control with immersive VR technology compared with traditional distraction methods. The sensation of going into the virtual world enhances a patient’s presence in the environment and draws attention away from pain.^8^ As immersion increases, so does pain reduction. This is likely because an individual’s attention is less available for pain perception.^9^ Virtual reality is particularly engaging for children because they often become engrossed in imaginative play.^10^ Systematic reviews of distraction-based therapies have confirmed that low-technology options are less efficacious than high-technology ones.^11^^,^^12^ Studies related to procedural needle pain have used different VR sequences, with different resultant participant responses.^13^^,^^14^

Passive and interactive distracting activities, such as guided imagery and hypnosis, can be used in children of all ages and developmental levels.^3^ Hypnosis has historically been widely published as very effective for preprocedural anxiety and pain.^12^ Hypnosis is defined as “a state of consciousness involving focused attention and reduced peripheral awareness characterized by an enhanced capacity for response to suggestion.”^15^ Hypnosis helps patients focus their attention to lessen anxiety and pain.^15^ Prior research has shown that children over the age of 3 years respond to medical hypnosis and that hypnotic responsivity is generally greater in children than in adults.^16^^,^^17^ It has been proposed that hypnotherapy may be the preferred nonpharmacologic intervention for young children given how easily and fluidly they enter trance-like states (eg, playing with imaginary “friends”).^17^^,^^18^

Our primary objective was to assess whether VR hypnosis would result in lower procedural anxiety and/or pain scores than those at baseline for pediatric patients undergoing nonemergent needle-related procedures. Our secondary outcomes were to determine parental and child satisfaction with the VR-assisted procedure and assess the relationship of patient fatigue and hunger with their pain/anxiety.

## Methods

This study was a single-center, prospective cohort study. Eligible participants (aged 8-15 years inclusive) received virtual reality hypnotherapy (VRHT) and included anyone who presented to the emergency department (ED) requiring a needle procedure (intravenous cannulation or venesection). Children were excluded from the study if they did not speak English; were cognitively impaired; had a history of affective disorder, attention disorder, or psychotropic medication use; were deaf or blind; or were in severe distress and required immediate treatment. Patients were identified and eligibility assessed by physicians working in the ED using a convenience sampling approach. Informed parental (includes legal guardian) consent and child assent were obtained. Local ethics committee approval was obtained for this study (REC reference: GEN/695/18).

The Samsung Gear VR head-mounted display (HMD) with Samsung 7S and headphones delivered an immersive 360° video featuring a hypnotherapist for 10 minutes. The VR video took the patient through an induction process before the needle intervention using favorite place hypnotic induction and the switches method with suggestions of hypnoanxiolysis and analgesia.^19^, ^20^ The video played for at least 6 minutes to allow for maximum potential trance to be achieved before the needle procedure. The use of a local vapocoolant spray or topical anesthetic cream was left to the discretion of the proceduralist.

Measurements were taken twice: at the baseline before the child wore the HMD and at 2 minutes after the intervention was finished, that is, cannula was in situ or plaster applied. The Visual Analog Scale (VAS; 10 cm horizontal) for self-reported anticipatory anxiety was marked by the child when asked “how afraid are you of the blood being taken?” After the procedure, children were then asked “what was the most anxiety/stress/worry felt during the procedure?” Parents marked on VAS their child’s anxiety before and during the procedure. They then marked down their own anxiety scores on a similar VAS. The worst pain was assessed reflecting the maximal pain intensity experienced by the participant during the procedure. We used the 10-cm Color Analog Scale (CAS).^21^, ^22^ At the conclusion of the intervention, the parent, child, and proceduralist rated how easy they believed that the needle procedure was on a 5-point Likert scale (from “very easy” to “very hard”).

### Statistical Analyses

Data entry included demographic data such as age and sex. Confounding variables included the following: grade of the proceduralist, protocol variations, concurrent analgesia usage, procedural success, number of attempts, and patient fatigue and hunger. We used IBM SPSS Statistics for Windows, version 26, for data analysis. After considering a Cochrane review that assessed anxiety and pain for needle-related procedures in children, in particular randomized controlled trials assessing VR as distraction,^12^ a sample of 25 (20+5) was selected, allowing for a 25% dropout rate. In our analysis, we used frequencies for descriptive purposes. Medians and interquartile ranges (IQRs) were chosen for all continuous variables. When relationships between the variables were assessed, the nonparametric tests included Spearman correlation coefficient for continuous variables, Chi-squared test or Fischer Exact test for categorical variables, and Mann-Whitney tests for categorical and continuous variables. Pain and anxiety were analyzed as a change from baseline scores and percentage change from the baseline group mean.

## Results

### Characteristics

In total, 22 participants with a median age of 10.5 years (IQR, 9-12 years) were recruited (Table 1). Nine (41%) patients received a local vapocoolant spray, 1 received a topical anesthetic cream, and 12 (55%) received neither. With missing values for 3 participants, 7 (37%) participants reported keeping their eyes open while wearing the HMD, 6 (32%) reported a mixture of eyes being both open and closed, and 6 (32%) kept their eyes closed throughout hypnotic induction.

**Table 1:** Group Characteristics

Characteristics	Total (N=22)	Total frequency (%)
Participants: female sex	12	55
Parents: female sex	21	95
Successful needle procedures	19	86
IV cannulation	17	78
Venesection	5	22
Oral analgesia within 6 hours	9 (missing, n=6)	56
Kept headset in place for the entire procedure	17	78

IV, intravenous.

### Anxiety and Pain

The change in patient anxiety and pain scores for the individuals are demonstrated in Table 2. The change in patient anxiety had a strong association with the change in parental perception of the participants’ anxiety (*R*_*S*_=0.61; *P*=.004). Similarly, the change in parental perception of the participants’ pain showed a very strong association with the change in the participants’ pain (*R*_*S*_=0.83; *P*<.001). A significant association was identified between local anesthetic/vapocoolant use and parental perception of the participants’ anxiety (*U*=15; *Z*=−2.82; *P*=.004). However, there was no significant association between topical anesthetic/vapocoolant use and any of the measured pain scores. Timely oral analgesia usage was associated with a significantly lower participant pain scores (*U*=8; *Z*=−2.49; *P*=.012) during the procedure. With regard to whether the participants kept the HMD in place during the entire procedure, it was associated with a decreased change in subjective pain (*U*=13; *Z*=−2.31; *P*=.019) and decreased change in parental perception of the participants’ pain (*U*=13.5; *Z*=−2.19; *P*=.025).

**Table 2:** Fear/Anxiety and Pain Scores Associated With Painful Procedure

Variable name	Preprocedural baseline score, median (IQR)	Max reported score, median (IQR)	Change from median baseline, median (IQR)	Change from median baseline (%)
Participant anxiety	2.40 (0.75-5.43)	2.73 (0.88-4.25)	−0.23 (−2.96 to 2.48)	−9.58
Parental perception of participant anxiety	3.50 (1.55-7.43)	1.80 (0.15-5.00)	−1.50 (−4.35 to 0.60)	−42.86
Participant pain	3.00 (0.18-5.83)	2.70 (1.55-7.13)	0.50 (−2.63 to 3.20)	16.67
Parental perception of participant pain	4.15 (0.42-6.20)	1.75 (0.43-4.23)	−0.90 (−4.13 to 1.03)	−21.69
Parental anxiety	1.46 (0.28-4.30)	1.10 (0.10-4.93)	0.00 (−2.73 to 1.35)	0.00

IQR, interquartile range; max, maximum.

### Satisfaction

Eleven of the participants reported that that the needle intervention was “easy” or “very easy.” Seven reported it as “average,” and 4 reported that it was “hard” or “very hard.” Nineteen (85%) of physician (proceduralists) reports stated that the needle intervention was “easy” or “very easy,” and the remaining 3 reports stated that it was “average.” Parents reported similar satisfaction scores, with 19 (85%) reporting that the needle intervention was “easy” or “very easy,” 2 reported that it was “average,” and 1 reported that it was “very hard.”

## Discussion

This study determined the potential influence of VRHT for pediatric needle-related procedures in an ED setting on anxiety and pain scores. This innovative use of VR technology demonstrates that hypnoanxiolysis, without the physical requirement of an on-site hypnotherapist, benefits pediatric patients undergoing needle-related procedures in an ED setting.

The change in the participants’ anxiety scores showed that most of the participants reported a reduction in anxiety from the baseline (median, −0.23; IQR, −2.96 to 2.48) during the needle-related procedures. Parental perception of the participants’ anxiety was also reduced (median, −1.5; IQR −4.35 to 0.6). We subjectively observed that some children were more anxious than they self-reported, and parental perceptions of anxiety scores may have been a more accurate measure for these cases. In a similarly designed study using a 100-mm VAS for anxiety, the VR group reported significantly lower stress intensity than the controls.^23^ Our study’s procedural median anxiety (2.73 cm) scores were better than this equivalent control group’s (41 mm) with a similarly recruited age profile and painful procedure (venipuncture).

The change in the participants’ median pain scores were slightly increased as related to the needle procedure (median, 0.5; IQR, −2.63 to 3.2). This was not unexpected. A study identified a minimum clinically significant difference using a CAS of 1 out of 10.^21^ This estimate was stable for children with moderate-to-severe pain, irrespective of age, sex, and ethnicity.^24^ However, no minimum clinically significant difference was established for children with mild baseline pain experiencing procedural needle pain in the emergency setting. In our study, the median self-reported change in pain was 0.5 out of 10 using CAS during the needle procedure. Interestingly, greater than half of the parents perceived an actual reduction from the baseline pain for their children (median, −0.9; IQR, −4.13 to 1.03). This identified to the study team that VRHT may have potential as an analgesic intervention for pre-existing mildly painful conditions. Another VR study analyzed pain scores in a similar population using a 100-mm VAS after venepuncture only.^23^ The standard-of-care control group did not use any distraction techniques. This study’s VR group reported significantly lower pain intensity than the controls.^23^ Our study reports lower pain scores than the aforementioned study’s control group with a similar recruited age profile and painful procedure (venipuncture). Interestingly, with respect to analgesia optimization, the use of topical anesthetic/vapocoolant was associated with a decrease in the participants’ anxiety scores but not with a change in the participants’ pain scores. Timely effective oral analgesia before the procedure was associated with significantly lower participant pain scores (*U*=8; *Z*=−2.49; *P*=.012) during the procedure. However, because of missing data (n=6), this was a small sample size, with only 9 of 16 (56%) patients receiving timely oral analgesia and 12 of 22 (55%) receiving no topical anesthetic agents.

This pilot cohort study had several limitations. The patient cohort was a slightly heterogeneous group reflective of individual ED presentations that were recruited opportunistically. There was no control group used in this pilot study, which is essential to further evaluate the mechanism of action, degree of anxiolysis, and pain minimization. Convenience sampling was used because of resource limitations. We did not assess the effects of patient diagnosis, for example, presence or absence of sickle cell disease, length of time spent in the ED, or location of needle procedure, in this study. Most parents were women, so the effects of parental sex on the participants’ pain or anxiety were not assessed. Topical and oral analgesic usage was nonuniformly applied, which reflects common local practice. The full degree of the effect of interplay between participant sex, fatigue, hunger, fear, pain, and previous pain experiences on scores is beyond the design of this study to evaluate. Parental perceptions of pain and anxiety during the procedure may have been impaired by the inability to see their child’s face while wearing the HMD. Clinically, we found that some children had an excellent response, showing signs of deep relaxation/hypnotic trace; however, trying to isolate which patients were more compatible with virtual hypnotherapy was beyond the scope of this study. Ideally, one would formally assess the “hypnotizability” of an individual to determine their suitability before implementing a hypnotic intervention.

## Conclusions

This was a prospective interventional pilot study involving children aged 8-16 years undergoing a needle-related procedure in the ED. We used VR to deliver hypnotherapy, with successful minimization of pain and reduction in anxiety in this population, proving the efficacy of this novel therapy. This is the first study using VRHT in an ED setting. This technology is feasible to deliver in the ED because it is nonexpensive and does not require any additional training or personnel. Future steps could include a randomized controlled trial to evaluate the comparative effectiveness of VRHT on pediatric anxiety and pain in the acute setting.

## Potential Competing Interests

The authors report no competing interests.
